# Soil bacterial and fungal diversity and composition respond differently to desertified system restoration

**DOI:** 10.1371/journal.pone.0309188

**Published:** 2025-01-06

**Authors:** Chengchen Pan, Feng Yuan, Yaling Liu, Xiaoya Yu, Jiliang Liu

**Affiliations:** 1 Northwest Institute of Eco-Environment and Resources, Chinese Academy of Sciences, Lanzhou, China; 2 National Center of Pratacultural Technolgoy Innovation (Under Preparation), Hohhot, China; 3 School of Tourism and Resource Environment, Qiannan Normal University for Nationalities, Duyun, China; JKI: Julius Kuhn-Institut Bundesforschungsinstitut fur Kulturpflanzen, GERMANY

## Abstract

Desertification is a major ecological issue worldwide that results in the destruction of terrestrial ecosystems. Restoration of desertified ecosystems has been carried out in recent decades, but the role of soil microorganisms in this process is poorly understood. Thus, to deconstruct the effects of desertified system restoration on soil microbial communities, we examined the changes in soil characteristics as well as the variations in and drivers of soil microbial diversity and community composition of the Hulun Buir Sandy Land in Northeast China, where restoration activities have been performed for approximately 30 years. The results revealed that with desertified system restoration, plant species richness and aboveground biomass increased significantly. The soil properties, characterized by organic carbon, total nitrogen and available nitrogen content improved. Moreover, soil pH decreased significantly from 7.75 in mobile dunes to 7.17 in fixed dunes (*P* < 0.05). Compared to mobile dunes, the Chao1 and Shannon diversity indices of bacteria increased significantly in fixed dunes. In contrast, the fungal richness index (Chao1 index) decreased significantly during desertified system restoration. The fungal Shannon diversity index also showed a decreasing trend, although it was not significant (*P* > 0.05). Proteobacteria was the most prevalent bacterial phylum, with a relative abundance of over 40%. In fixed dunes, the relative abundances of Actinobacteria, Acidobacteria, Gemmatimonadetes, and Chloroflexi significantly increased, whereas the relative abundance of Firmicutes significantly decreased. For fungi, Ascomycota was the dominant phylum, with a relative abundance of 97.6% in fixed dunes compared with 82.4% in mobile dunes. Plant species richness and soil pH were the major determinants of the soil microbial communities. This research provides important insights into the changes in soil microbial communities and their relationships with environmental factors during desertified system restoration, which can help guide sustainable land management practices and the restoration of desertified areas.

## Introduction

Desertification is a major ecological issue worldwide, that destroys the stability and functionality of terrestrial ecosystems [1]. It is estimated that over 3.6 billion hectares of land, which constitute approximately one-fourth of the Earth’s total land area, have affected by desertification [2]. The restoration of desertified areas has immense significance in reversing these detrimental trends. To combat desertification, many measures, such as afforestation, fence enclosures, and improvement in land management, have been adopted to restore affected areas and protect the balance and stability of ecosystems. The research on desertified area restoration has been focused mainly on the studies of soil and vegetation [3]. Soil, as the foundation of ecological stability, and vegetation, as indicators of ecological recovery, have received extensive attention [4, 5]. In general, the restoration of desertified landscapes often leads to remarkable improvements in vegetation characteristics including increased plant species diversity, as well as increased vegetation cover and biomass [4]. This not only contributes to the aesthetic appeal of the environment but also plays a crucial role in soil stabilization, preventing erosion and promoting nutrient cycling. Therefore, the process of desertified system restoration has the potential to provide substantial benefits for both vegetation and soil, offering hope for the rehabilitation of degraded landscapes and the mitigation of ecological imbalances. Recent studies have highlighted the importance of soil microorganisms in this endeavor [6]. However, our understanding of the role of microorganisms in restoring desertified areas is still limited.

Soil microorganisms play a crucial role in the relationship between vegetation and soil [7, 8]. They contribute significantly to the decomposition of organic matter, nutrient cycling, and soil structure formation [9, 10]. Microorganisms interact closely with plant roots, forming symbiotic relationships such as mycorrhizal associations that increase plant nutrient uptake [11]. Vegetation type and richness also influence the composition of soil microbial communities [12–15]. A wide range of biotic and abiotic factors can influence soil microorganisms, such as soil pH [16], nutrient availability [17], moisture [18], organic matter [19], and vegetation type and diversity [20, 21]. Understanding the complex interactions that occur between soil microorganisms and vegetation–soil systems is essential for sustainable land management and ecological restoration [22, 23].

Bacteria and fungi are two major components of soil microbial communities. Bacteria are actively involved in the decomposition of organic matter and nutrient cycling [24]. For example, some bacteria can fix nitrogen, converting it into a form that plants can utilize, which is essential for plant growth in restored areas [25]. On the other hand, fungi are eukaryotic organisms with a network of hyphae [26]. They are important in decomposing complex organic matter and have an extensive hyphal network that can explore a larger soil volume [27]. This characteristic enables fungi to access and break down complex substrates that are resistant to bacterial decomposition [27]. In the context of desertified system restoration, fungi can contribute to the long term stabilization of soil structure [28]. They can bind soil particles together, improving soil porosity and water holding capacity, which is crucial for preventing soil erosion and promoting the growth of vegetation in desertified areas [29].

China is among the countries most severely affected by desertification. Desertification has brought about numerous challenges for ecological balance, agricultural production, and sustainable development in the country. According to the latest statistics and research, approximately 168.68 million square kilometers of land in China are estimated to be affected by desertification [30]. The Hulun Buir Grassland is an important ecological barrier in northern China. Owing to factors such as drought and land reclamation for human activities, problems related to desertification in the Hulun Buir Grassland in the Inner Mongolia Autonomous Region have become increasingly severe [31]. Three large sand belts have emerged, constituting the Hulun Buir Sandy Land with a total area of approximately 880,000 hectares [32]. It is extremely urgent to control sand movement in this ecosystem. Systematic restoration efforts have been performed in the Hulun Buir Sandy Land for approximately 30 years [33]. However, our understanding of the influence of desertified system restoration on soil microbial community diversity and composition remains limited.

Here, the variations in bacterial and fungal diversity and composition were examined to evaluate the effectiveness of desertified system restoration (fencing) in the Hulun Buir Sandy Land in China. We aimed (1) to examine the response of the diversity and composition of the soil microbial communities to desertified system restoration; and (2) to analyze the factors driving the soil bacterial and fungal community structure. The following two hypotheses were examined: (1) desertified land restoration significantly enhances vegetation characteristics and decreases soil pH, which control the soil microbial community structure; and (2) the structure of the soil bacterial and fungal communities respond to desertified system restoration.

## Materials and methods

### Ethics statement

All examined samples were collected on sandy land and hence no specific permits were required for the field studies. The field studies did not involve endangered or protected species.

### Site selection

**Fig 1:**
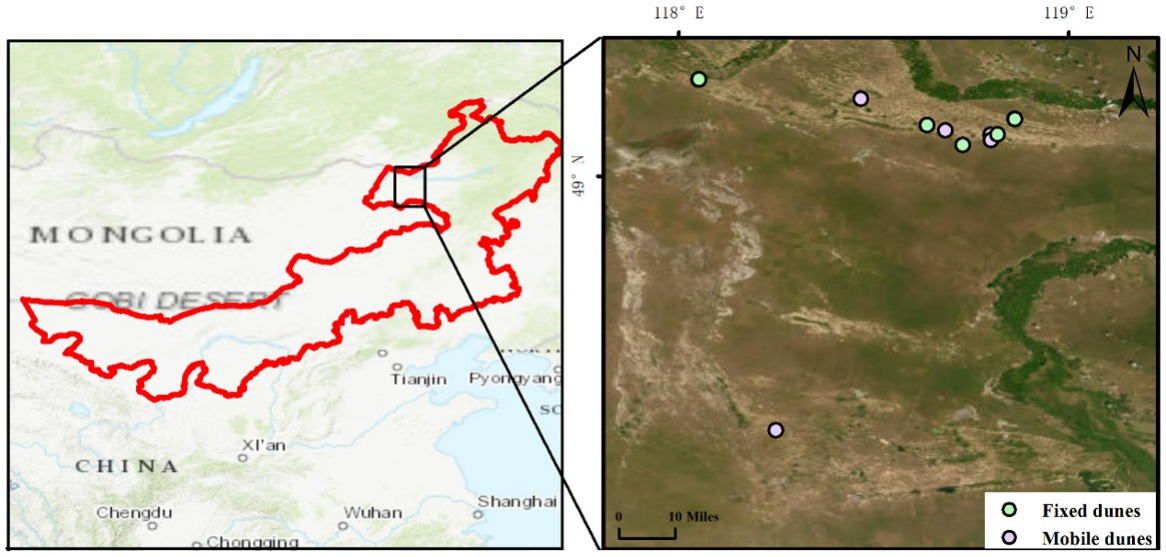
Map showing the location of the sampling sites of the Hulun Buir Sandy Land in China in Inner Mongolia, China.

The field work was carried out in Hulun Buir Sandy Land (48º20.0′ N to 49º14.0′ N, 118º3.0′ E to 118º51.0′ E), which is located in New Barag Left Banner and Chen Barag Banner, Inner Mongolia Autonomous Region of northeastern China (Fig 1). This region is characterized by a temperate semiarid continental climate, with an annual mean temperature (MAT) of -0.85–-0.1°C and a mean annual precipitation (MAP) of 280–330 mm. MAT and MAP data were obtained from the WorldClim database on the basis of the sampling site coordinates (www.worldclim.org) [34]. The soil is dominated by sandy chestnut soil and is prone to wind erosion. To combat desertification, sand-fixing vegetation has been established in this region. Fixed, nongrazed dunes and mobile dunes were selected as representative restoration areas. All the selected fixed dunes were restored from mobile dunes after 30 years of fencing. Before fencing, the landscape was characterized as mobile (with vegetation coverage < 5%) sandy land. The vegetation in the fixed dunes was dominated by *Artemisia halodendron*, *Caragana microphylla*, and *Corethrodendron fruticosum*, and these plant species had more than 60% vegetation coverage.

### Soil sampling and vegetation survey

Field sampling was conducted in early August 2018, during the season with the highest vegetation productivity. A total of 15 pairs of mobile and fixed dunes were selected. All the selected sites were at least 100 m apart from each other. Thirty plots (1×1 m^2^) were randomly established. In each plot, 5 soil core samples (3.5 cm in diameter) were randomly collected at a depth of 0–10 cm and combined. The composite soil samples were sieved (2 mm) immediately and divided into two parts in the field. One part was stored in a portable refrigerator and then kept at -20 °C in the laboratory for further molecular analysis; the other part was air-dried for the determination of edaphic properties (pH, soil organic carbon (SOC), total nitrogen (TN), available nitrogen (AN)).

SOC was determined via the K_2_Cr_2_O7-H_2_SO_4_ oxidation method [35]. TN was determined via an elemental analyzer (VarioMacro Cube Elementar, Germany) [36]. The soil NH_4_^+^-N and NO_3_-N contents were determined via KCl extraction and a continuous flow analyzer (Futura, Alliance, Frépillon, France) [36]. AN was calculated as the sum of soil NH_4_^+^-N and NO_3_-N. pH was measured in 1:2.5 soil:water extracts [37].

In each plot, all living plant species were identified at the species level and clipped for aboveground biomass (PB) measurements. All the samples were oven-dried at 65 °C for 48 h until their weights did not change. The plant species richness (PR) was estimated by the total number of species.

### Soil microbial DNA extraction, PCR and high-throughput sequencing

The soil microbial DNA from each sample was extracted via a magnetic bead DNA extraction kit (Tiangen Biochemical Technology Co. Ltd., Beijing, China). After PCR amplification, the bacterial and fungal communities were examined using the V3–V4 hypervariable regions of the 16SrRNA genes with the primers 341F (CCTAYGGGRBGCASCAG) and 806R (GGACTACNNGGGTATCTAAT) [38], and ITS1-5F genes with primers 1737F (GGAAGTAAAAGTCGTAACAAGG) and 2043R (GCTGCGTTCTTCATCGATGC) [39], respectively. PCR was performed in a 30-μl mixture containing 15 μL of Phusion^®^ High-Fidelity PCR Master Mix (New England Biolabs), 1 μl of each primer (10 μM), and ~ 10 ng of template DNA (gDNA). Thermal cycling for both bacteria and fungi was conducted with an initial denaturation step at 95 °C for 5 min, followed by 34 cycles at 91 °C for 1 min, 57 °C for 45 s, 72 °C for 1 min, and finally at 72 °C for 10 min. PCR products were extracted from 2% agarose gels and purified using the GeneJET gel extraction kit (Thermo Fisher Scientific) according to the manufacturer’s instructions. An Ion Plus Fragment Library Kit 48 rxns (Thermo Fisher Scientific, Wilmington, DE, USA) was used to generate the sequencing library. The bacterial 16S rRNA and fungal ITS genes were sequenced using Ion S5 XL platform (Thermo Fisher Scientific) with single-end reads at Novogene Bioinformatics Technology Co. Ltd., Beijing, China [40]. All raw bacterial and fungal sequences have been deposited at the National Center for Biotechnology Information (NCBI) Sequence Read Archive (SRA) with accession numbers PRJNA667483 (bacteria) and PRJNA1130371 (fungi), respectively.

### Processing of sequencing data

The raw sequences were first trimmed using Cutadapt (version 1.9.1) to remove short and low-quality sequences [41]. Chimera removal was conducted with the UCHIME algorithm [42]. The high-quality sequences were clustered into operational taxonomic units (OTUs) at the 97% similarity level using UPARSE. OTUs containing fewer than 3 reads were removed to avoid possible biases. Then, each sample was rarefied to 51109 and 71156 bacterial and fungal reads, respectively, from the high quality sequences for both alpha-diversity and beta-diversity analyses. The representative sequences for the remaining OTUs were assigned on the basis of the bacterial SILVA [43] (version 132) database and fungal UNITE [44] (version 8.0) database.

### Statistical analysis

The effects of desertified system restoration on vegetation and soil properties and microbial alpha-diversity, characterized by richness (Chao 1) and diversity (Shannon) indices [45], were examined using two independent sample t-tests. The relationships between environmental variables and microbial alpha-diversity were inspected by Pearson’s correlation analysis.

All of the variations in the soil microbial communities were estimated on the basis of the OTU matrix. Nonmetric multidimensional scaling (NMDS) ordination plots were used to visualize the structure among the samples based on the Euclidean distance in the OTU matrix. Analysis of similarity (ANOSIM) [46], the multiresponse permutation procedure (MRPP), and permutational multivariate analysis of variance (PERMANOVA) were also conducted to test the significance of variations among bacterial communities [47, 48]. These statistical analyses were implemented in the vegan R package [49].

Canonical correlation analysis (CCA) was conducted to elucidate the relationships between microbial groups and explanatory variables, including edaphic and vegetation factors. The “forward selection” procedure was performed to select driving factors using Monte Carlo permutation (999 repetitions) for each dataset in the analysis. The entire process was performed using trial version CANOCO 5.0 [50].

## Results

### Soil physicochemical environment and vegetation characteristics

With desertified system restoration, plant species richness (PR) and aboveground biomass (PB) increased significantly. The soil properties of the fixed dunes differed from those of the mobile dunes (Table 1; S1 Table). The soil organic carbon (SOC), soil total nitrogen (TN), soil ammonium nitrogen (NH_4_^+^-N), soil nitrate nitrogen (NO_3_^*˗*^-N) and clay contents in the fixed dunes were significantly greater than those in the mobile dunes (*P* < 0.05). However, pH decreased significantly after the dunes were fixed (*P* < 0.05).

**Table 1:** The vegetation and soil properties of mobile and fixed dunes.

	Mobile dunes	Fixed dunes
pH	7.75 ± 0.12a	7.17 ± 0.09b
NH_4_^+^-N (mg kg^-1^)	4.79 ± 0.13b	6.77 ± 0.29a
NO_3_^*˗*^-N (mg kg^-1^)	2.44 ± 0.07b	5.63 ± 0.54a
SOC (g kg^-1^)	0.90 ± 0.05b	4.63 ± 0.70a
TN (g kg^-1^)	0.14 ± 0.01b	0.58 ± 0.13a
Clay (%)	5.33 ± 0.59b	12.72 ± 6.58a
PB (g/m^2^)	4.94 ± 1.76b	27.60 ± 1.81a
PR (species/m^2^)	1.03 ± 0.25b	6.83 ± 0.39a

Note: NH_4_^+^-N, NO_3_^*˗*^-N, SOC, TN represents the abbreviations of soil ammonium nitrogen, soil nitrate nitrogen, soil organic carbon, soil total nitrogen, while PB and PR represents plant aboveground biomass and plant species richness, respectively. Values are the means ± SE (n = 15). Different letters indicate significant differences between both grassland types (P < 0.05).

### Diversity patterns of the soil bacterial and fungal communities

**Fig 2:**
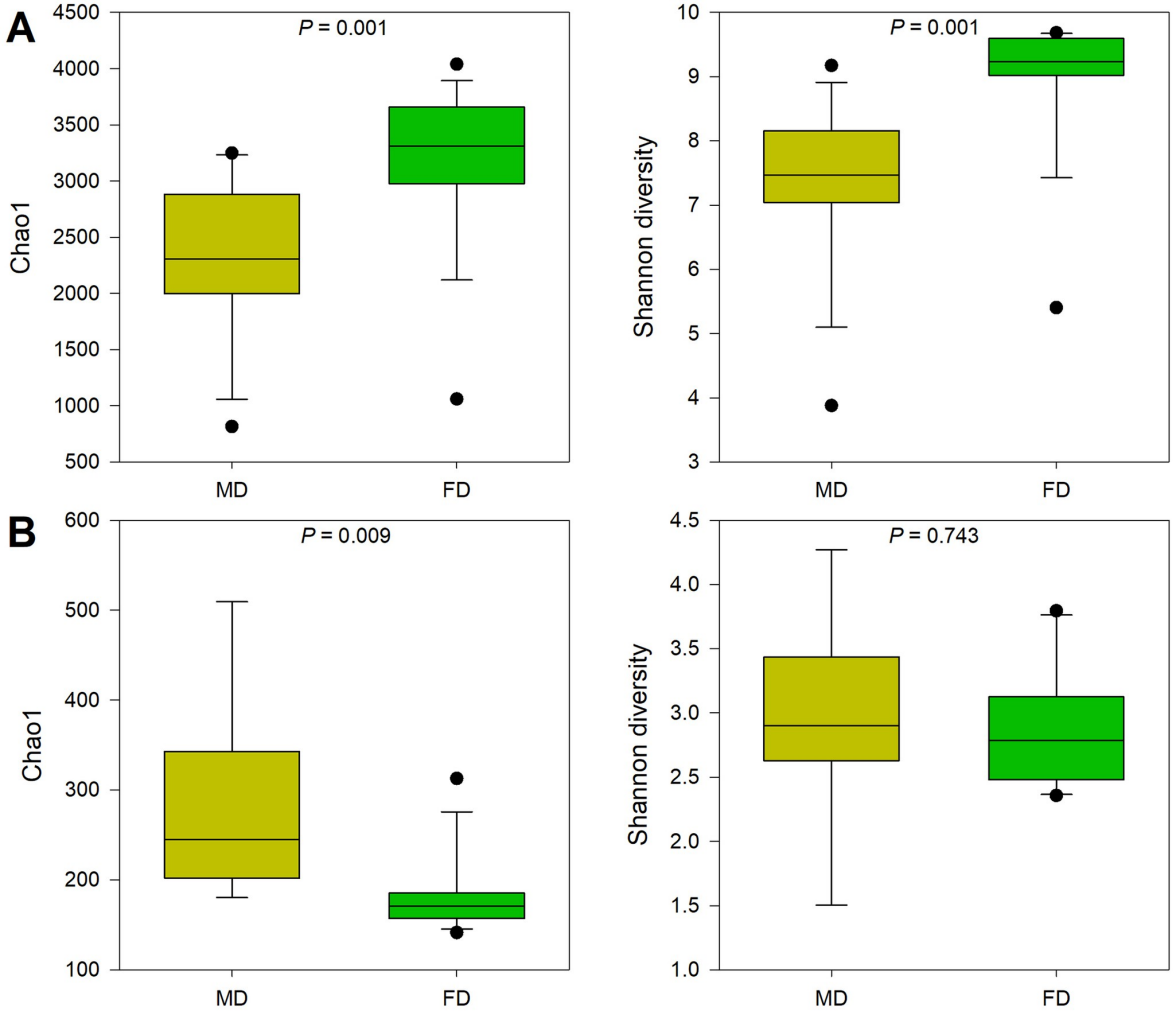
Soil bacterial (A) and fungal (B) alpha-diversity indices in mobile and fixed dunes. The alpha diversity indices were based on the OTU richness (Chao 1 index) and diversity (Shannon diversity) indices.

The patterns of bacterial and fungal diversity varied with the degree of desertified land restoration, but the trends were inconsistent (Fig 2; S2 Table). Compared with those of mobile dunes, the Chao1 and Shannon diversity indices of bacteria increased significantly. The variation in fungal diversity indices presented a trend opposite to that of bacteria. The fungal richness index (Chao1 index) decreased significantly during the restoration of desertified land. A similar decreasing trend was also observed in the fungal Shannon diversity index; however, the variation was not significant (*P* > 0.05).

### Changes in the compositions of the soil bacterial and fungal communities

**Fig 3:**
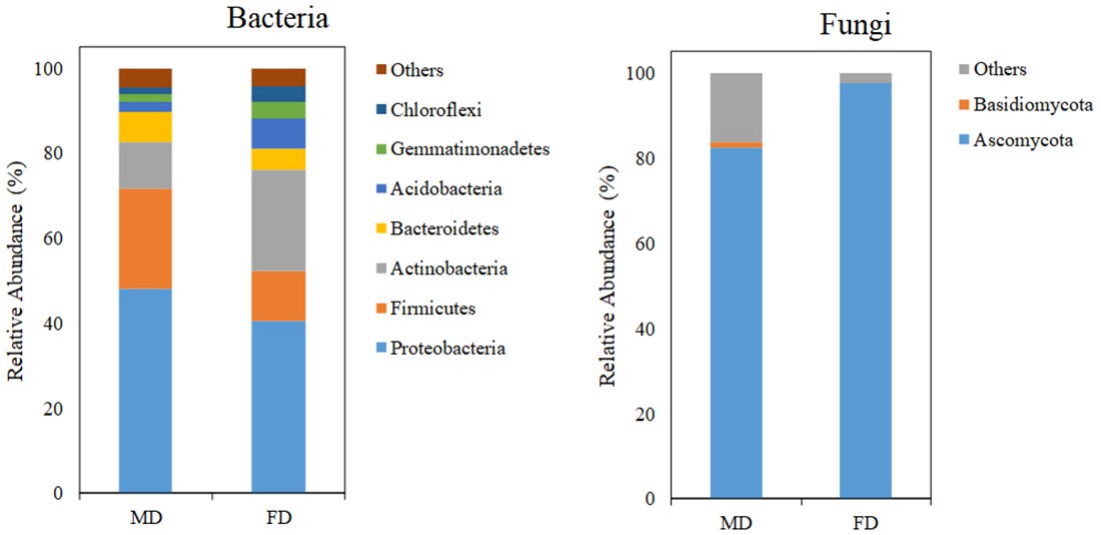
Relative abundances of dominant phyla in soil bacteria (a) and fungi (b) during restoration. Note: “Others” represents all the phyla with a relative abundance <1%.

The main bacterial phyla with a relative abundance greater than 1% in both dune types were: Proteobacteria, Firmicutes, Actinobacteria, Bacteroidetes, Acidobacteria, Gemmatimonadetes, and Chloroflexi (Fig 3; S3 Table). Among them, Proteobacteria was the most prevalent bacterial phylum, and its relative abundance was greater than 40%. The relative abundances of Actinobacteria, Acidobacteria, Gemmatimonadetes, and Chloroflexi significantly increased (*P* < 0.05), whereas that of Firmicutes significantly decreased in the fixed dunes (*P* = 0.036; Fig 3).

For fungi, Ascomycota was the dominant phylum (Fig 3; S4 Table). The relative abundance of Ascomycota was greater in fixed dunes (accounting for 97.6% of the total sequences) than in mobile dunes (accounting for 82.4%).

**Fig 4:**
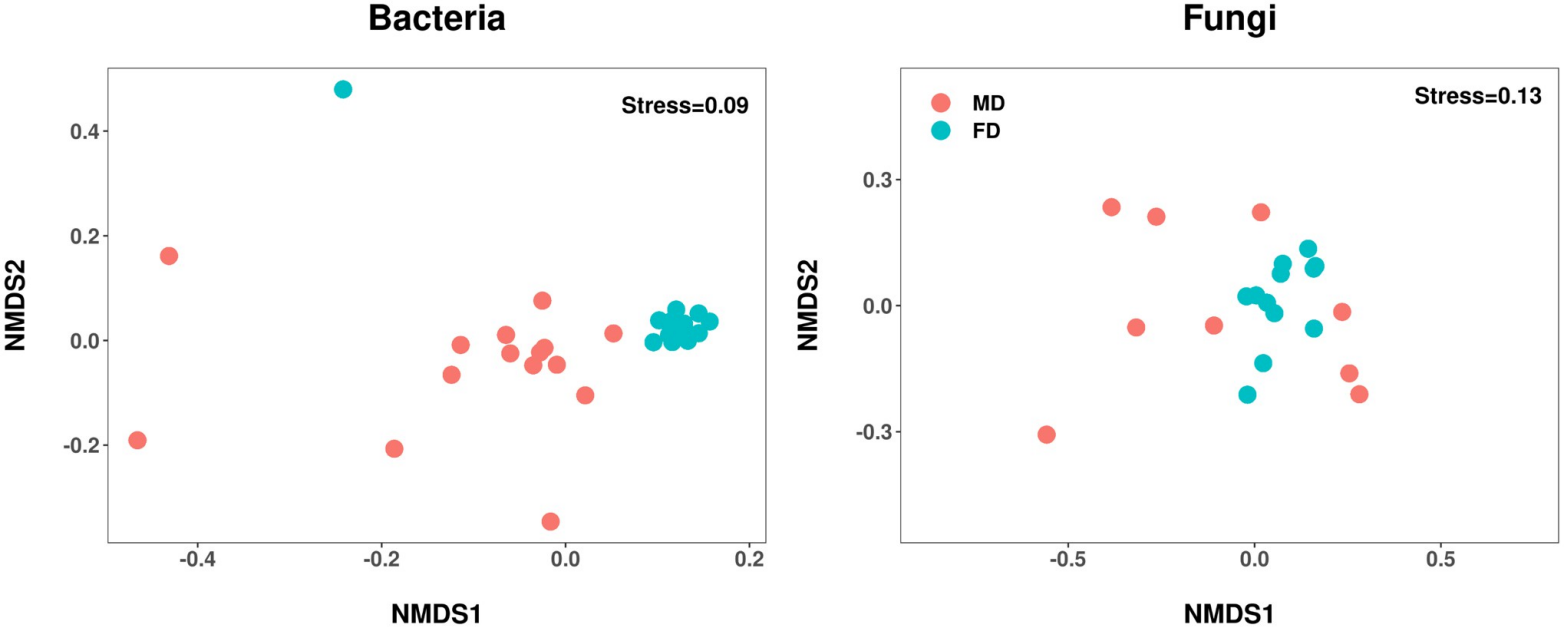
Nonmetric multidimensional scaling (NMDS) ordination plot based on the Bray‒Curtis distance of samples for the bacterial (a) and fungal communities (b) in the mobile and fixed dunes. MD, mobile dunes; FD, fixed dunes.

Similarly, the soil bacterial and fungal community structures changed significantly with vegetation restoration (*R*^2^ = 0.233, *P* = 0.001; *R*^2^ = 0.161, *P* = 0.001; Fig 4). Furthermore, nonmetric multidimensional scaling analysis (NMDS) was carried out to assess microbial beta diversity. The NMDS plots clearly separated the soil bacterial and fungal communities between the mobile and fixed dunes. ADONIS, ANOSIM, and MRPP also revealed that the soil bacterial and fungal community structures differed significantly during the restoration of desertified land (Table 2, *P* < 0.05).

**Table 2:** Statistical analysis of differences in the soil bacterial and fungal communities between mobile and fixed dunes.

Items	Adonis		MRPP		Anosim	
	*R* ^2^	*P*	Delta	*P*	R	*P*
bacteria	0.76893	<0.001*	0.5722	0.001	0.4719	0.001
fungi	0.83185	0.004	0.4672	0.006	0.2712	0.005

### Relationships between microbial community composition and environmental factors

**Fig 5:**
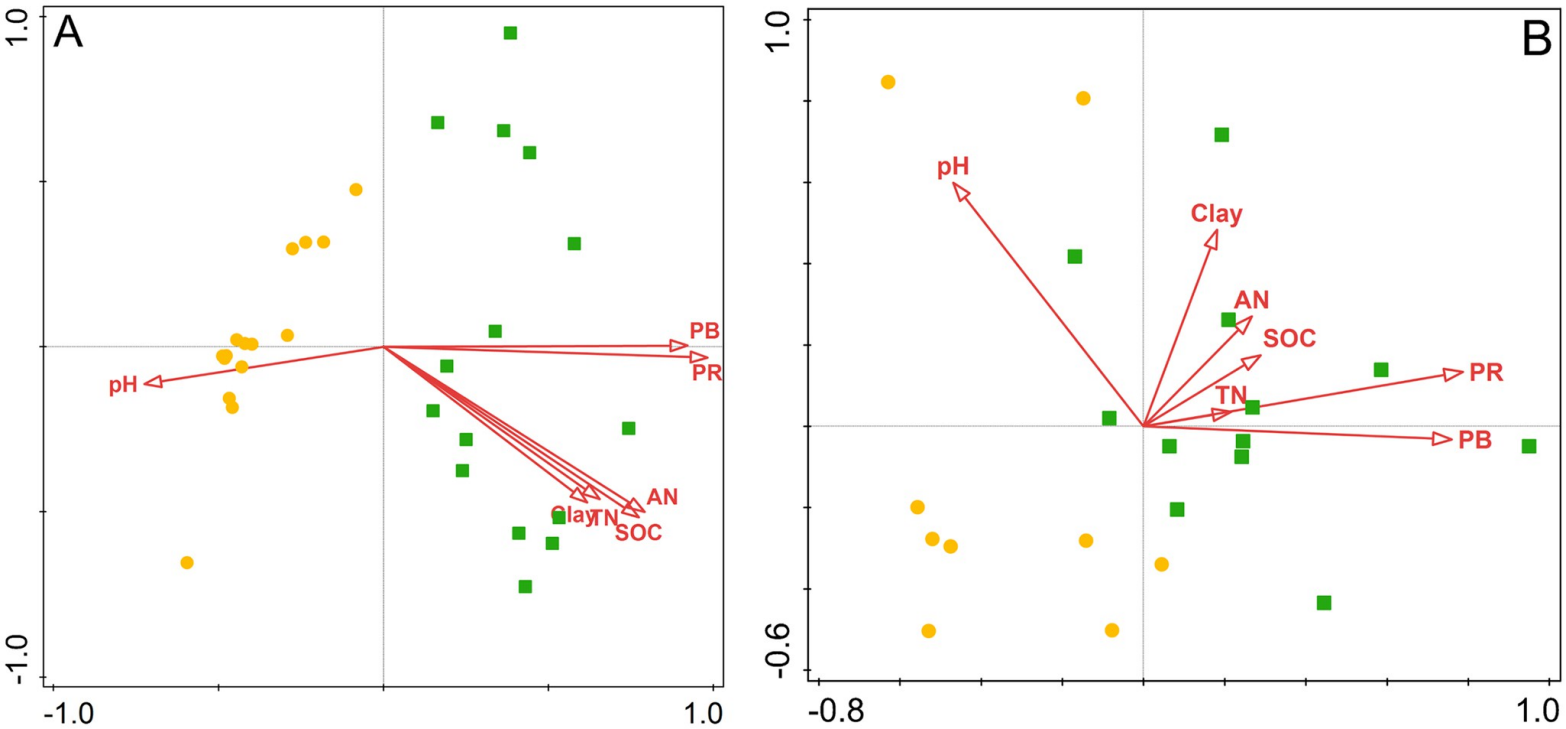
Biplot of the first two CCA axes showing the relationships between soil bacterial (A) and fungal (B) communities with environmental variables.

The effects of environmental factors on microbial communities were analyzed using CCA. The first two canonical axes explained 12.8% and 35.4% of the variation in bacterial and fungal phyla, respectively (Fig 5). The CCA model selection procedure revealed that PR and pH were the most significant factors driving the soil bacterial and fungal communities (*P* < 0.05).

## Discussion

### Changes in soil physicochemical and vegetation properties during desertified system restoration

The restoration of desertified landscapes is a critical practice with profound implications for ecological stability and environmental health.

The results of this study support the first hypothesis. Our results revealed that plant species richness and aboveground biomass increased significantly during desertified system restoration. An increase in plant species not only enriches the ecological structure but also enhances the resilience and functionality of an ecosystem [51]. An increase in biomass leads to a substantial accumulation of litter and contributes to carbon sequestration [4, 52].

The substantial increase observed in the content of clay particles is in agreement with the findings of Su et al. [53] and Yuan et al. [54], suggesting that restoration efforts can effectively modify soil texture and improve its physical properties. With respect to soil nutrients, a significant increase in organic carbon, total nitrogen, and available nitrogen occurred, which is in agreement with the reports of Li et al. [55], Zhang et al. [56] and Li et al. [57]. These enhancements in soil fertility are crucial for providing favorable environments for plant growth and metabolic processes. The increased plant species richness and biomass, along with the improved soil texture and nutrient status, collectively suggest that the restoration strategies implemented in our study were effective to a considerable extent. These positive changes contribute to the restoration and improvement of ecosystem health and functionality [58].

However, in contrast to some previous studies, we found a significant decrease in pH. For example, Zhang et al. [56] and Qi et al. [59] reported relatively stable or slight increases in pH values during restoration processes. The reasons for this discrepancy might be attributed to differences in soil types, initial soil conditions, geographical locations, and the specific restoration techniques employed. In this study, the decrease observed in soil pH might also be related to plant growth during desertified system restoration. The restoration of vegetation results in an increase in root exudates or litter input from plants and subsequently, an increase in the input of organic acids [60]. In such a situation in which, the input of organic acids increases, the soil pH tends to decrease [61].

### The impact of desertified system restoration on microbial diversity and composition

The present study revealed the diverse responses of bacterial and fungal communities to the restoration of desertified land, which does not support the second hypothesis. This finding is highly significant as it adds a substantial layer of complexity to our comprehension of ecological succession under harsh and challenging environments.

Our results revealed that the alpha diversity indices of bacteria significantly increased in fixed dunes in comparison with those in mobile dunes, contrary to the decreasing trend observed in the fungal diversity indices. This is partially in line with the findings of Sun et al. [62], who reported that soil bacterial and fungal communities presented distinct recovery patterns during ecosystem restoration. The significant increase in bacterial diversity may suggest that restoration provides improved niches and resources for bacterial growth and proliferation [63]. A more diverse and abundant bacterial community can enhance the decomposition of organic matter and nutrient cycling [64, 65].

Proteobacteria was the most abundant bacterial phylum in our study area, which is consistent with previous reports in similar ecosystems [21]. The significant increase in the relative abundances of Actinobacteria, Acidobacteria, Gemmatimonadetes, and Chloroflexi in fixed dunes indicated their potential role in the stabilization and improvement of soil conditions. These phyla are often associated with shifts in soil fertility and structure [66]. For example, Actinobacteria are known for their ability to produce antibiotics and enzymes that can break down complex organic compounds, contributing to nutrient availability [67]. Acidobacteria are involved in the decomposition of organic matter [68], whereas Gemmatimonadetes and Chloroflexi can play roles in carbon cycling and soil aggregation, respectively [69, 70]. In contrast, the higher relative abundance of Firmicutes in mobile dunes could be attributed to their adaptability to the harsher and more fluctuating conditions of these dunes [71]. Firmicutes are often associated with resistance to desiccation and extreme temperatures, allowing them to survive and persist in unstable dune environments [71].

For fungi, the dominance of Ascomycota and its greater abundance in fixed dunes than in mobile dunes aligns with the observations of Wang et al. [72]. Fungi play crucial roles in the decomposition of organic matter, in symbiotic relationships with plants, and in the formation of mycorrhizal associations that increase nutrient uptake [73]. The relatively high abundance of Ascomycota in fixed dunes could contribute to soil nutrient cycling and structural maintenance [74].

The changes in bacterial and fungal diversity and composition suggest a shift in ecological processes and functions with dune fixation. The increase in certain bacterial and fungal phyla in fixed dunes might contribute to nutrient cycling and soil organic matter decomposition, facilitating the establishment of a more stable ecosystem [75, 76]. This can lead to improved soil water holding capacity, increased soil porosity, and better soil aeration, all of which are essential for plant growth and the overall health of an ecosystem.

The present study revealed significant changes in bacterial and fungal community structures associated with vegetation restoration. Several previous studies have emphasized the intimate connection between vegetation and microbial communities [77, 78]. For example, Shanmugam and Kingery [79] demonstrated that alterations in plant composition can lead to corresponding shifts in microbial community structure. Understanding this connection helps in predicting and manipulating microbial communities for better ecosystem functioning.

NMDS, ADONIS, ANOSIM and MRPP analysis provided clear evidence of the differences in soil bacterial and fungal community structures between mobile and fixed dunes. Similar findings were reported in a study by Wang et al. [72], highlighting the profound impact of ecological restoration on soil microbial communities. These findings indicate that dune stability plays a crucial role in shaping microbial community composition. Developing targeted restoration strategies on the basis of dune type is crucial, as it helps in preserving and enhancing the unique microbial communities associated with each type of dune.

### Influence of environmental factors on the soil microbial community

Plant species richness was found to be the most important factor driving soil bacterial and fungal communities, suggesting the crucial role of plant diversity in shaping microbial communities [80]. This implies that maintaining a high level of plant diversity is crucial for fostering a balanced and functional soil microbial community. The mechanism underlying this relationship is likely complex. A rich plant community provides a diverse array of root exudates, which contain different compounds that selectively support the growth and metabolism of specific microbial taxa [81]. Additionally, different plant species create a mosaic of microenvironments within the soil, influencing factors such as soil moisture, aeration, and nutrient availability. This heterogeneity in the soil environment promotes the coexistence of a diverse range of bacterial and fungal species [82].

In addition to plant species richness, soil pH is an important variable driving soil microbial community structure. Previous research has elucidated the intricate relationship between soil pH and microbial communities in various contexts [83, 84]. Soil pH affects the availability of essential nutrients for microbial growth. At high pH values, nutrients such as nitrogen, phosphorus, and trace elements are less accessible to microorganisms due to changes in solubility and chemical forms [85]. Moreover, pH influences the charge and structure of soil colloids [86], which in turn affects the adsorption and desorption of nutrients and the ability of microorganisms to absorb them. The physiological and biochemical processes within microbial cells are also pH dependent. Different microbial species have specific pH optima for enzyme activities and metabolic pathways. Additionally, pH can influence competition and interaction among microbial species [87], as some species may be better adapted to certain pH conditions, thus altering community composition.

The recognition of plant species richness and pH as major determinants emphasizes the need for careful monitoring and management of these factors in restoration efforts. Maintaining a diverse plant community and optimal pH levels is essential for promoting a balanced and functional microbial community, which is critical for soil fertility and ecosystem resilience. A diverse microbial community associated with a rich plant community and suitable soil pH can more easily withstand environmental disturbances and adapt to changing conditions [88].

## Conclusions

In this study, we focused on the effects of desertified system restoration on the soil microbial community in the Hulun Buir Sandy Land, China. We found that desertified system restoration led to significant changes in both vegetation and soil properties. Compared with mobile dunes, plant species richness and aboveground biomass increased. With respect to soil characteristics, the soil organic carbon, total nitrogen, available nitrogen, and clay content increased in fixed dunes, soil pH decreased. The values of bacterial diversity indices, such as the Chao1 and Shannon indices, significantly increased, whereas the fungal diverisity indices exhibited the opposite trend. The bacterial and fungal community structures changed significantly. Environmental factors, namely, plant species richness and pH, were identified as the major determinants of the soil bacterial and fungal communities. Our study provides valuable insights for formulating effective restoration strategies and maintaining the long-term health of ecosystems. However, more studies are needed to further explore the complex interactions that occur between soil microorganisms and the environment in the context of desertification. This knowledge will be essential for sustainable land management and the successful restoration of desertified areas.

## Supporting information

S1 Table Detailed vegetation and soil properties of mobile and fixed dunes. (DOCX)

S2 Table Soil bacterial and fungal alpha-diversity indices in mobile and fixed dunes. The alpha diversity indices were based on the OTU richness (Chao 1 index) and diversity (Shannon diversity) indices.
(DOCX)

S3 Table Relative abundance greater than 1% of soil bacterial phyla in mobile and fixed dunes. (DOCX)

S4 Table Relative abundance greater than 1% of soil fungal phyla in mobile and fixed dunes. (DOCX)

## References

[1] D’Odorico P, Bhattachan A, Davis KF, Ravi S, Runyan CW (2013) Global desertification: Drivers and feedbacks. Adv Water Resour 51: 326–344.

[2] Dregne H, Kassas M, Rozanov B (1991) A new assessment of the world status of desertification. Desertification Control Bulletin 20: 6–18.

[3] Zhao H, Zhou R, Su Y, Zhang H, Zhao L, Drake S (2007) Shrub facilitation of desert land restoration in the Horqin Sand Land of Inner Mongolia. Ecol Eng 31: 1–8.

[4] Zuo X, Zhao H, Zhao X, Guo Y, Yun J, Wang S, et al. (2009) Vegetation pattern variation, soil degradation and their relationship along a grassland desertification gradient in Horqin Sandy Land, northern China. Environ Geol 58: 1227–1237.

[5] Li X, Jia X, Dong G (2006) Influence of desertification on vegetation pattern variations in the cold semi-arid grasslands of Qinghai-Tibet Plateau, North-west China. J Arid Environ 64: 505–522.

[6] Zheng W, Wu Q, Rao C, Chen X, Wang E, Liang X, et al. (2023) Characteristics and interactions of soil bacteria, phytocommunity and soil properties in rocky desertification ecosystems of Southwest China. Catena 220: 106731.

[7] van der Heijden MGA, Bardgett RD, van Straalen NM (2008) The unseen majority: soil microbes as drivers of plant diversity and productivity in terrestrial ecosystems. Ecol Lett 11: 296–310. doi: 10.1111/j.1461-0248.2007.01139.x 18047587

[8] Liu M, Li X, Zhu R, Chen N, Ding L, Chen C (2021) Vegetation richness, species identity and soil nutrients drive the shifts in soil bacterial communities during restoration process. Environ Mcirobiol 13: 411–424. doi: 10.1111/1758-2229.12913 33264476

[9] Sahu N, Vasu D, Sahu A, Lal N, Singh SK (2017) Strength of microbes in nutrient cycling: a key to soil health. In Meena V, Mishra P, Bisht J, Pattanayak A (eds) Agriculturally important microbes for sustainable agriculture. Singapore: Springer. 69–86.

[10] Prasad S, Malav LC, Choudhary J, Kannojiya S (2021) Soil microbiomes for healthy nutrient recycling in Current Trends in Microbial Biotechnology for Sustainable Agriculture. Singapore: Springer. 1–21.

[11] Marschner H, Dell B (1994) Nutrient uptake in mycorrhizal symbiosis. Plant Soil 159: 89–102.

[12] Han W, Wang G, Liu J, Ni J (2021) Effects of vegetation type, season, and soil properties on soil microbial community in subtropical forests. Appl Soil Ecol 158: 103813.

[13] Seitz VA, McGivern BB, Daly RA, Chaparro JM, Borton MA, Sheflin AM, et al. (2022) Variation in root exudate composition influences soil microbiome membership and function. Appl Environ Microb 88: e00226–22. doi: 10.1128/aem.00226-22 35536051 PMC9195941

[14] Chaparro JM, Sheflin AM, Manter DK, Vivanco JM (2012) Manipulating the soil microbiome to increase soil health and plant fertility. Biol Fert Soils 48: 489–499.

[15] Zhang N, Nunan N, Hirsch PR, Sun B, Zhou J, Liang Y (2021) Theory of microbial coexistence in promoting soil–plant ecosystem health. Biol Fert Soils 57: 897–911.

[16] Fierer N, Jackson RB (2006) The diversity and biogeography of soil bacterial communities. Proc Natl Acad Sci USA 103: 626–631. doi: 10.1073/pnas.0507535103 16407148 PMC1334650

[17] Koorem K, Gazol A, Oepik M, Moora M, Saks Ü, Uibopuu A, et al. (2014) Soil nutrient content influences the abundance of soil microbes but not plant biomass at the small-scale. Plos One 9: e91998. doi: 10.1371/journal.pone.0091998 24637633 PMC3956881

[18] Bastida F, Torres IF, Andrés‐Abellán M, Baldrian P, López‐Mondéjar R, Větrovský T, et al. (2017) Differential sensitivity of total and active soil microbial communities to drought and forest management. Global Change Biol 23: 4185–4203. doi: 10.1111/gcb.13790 28614633

[19] Tian J, He N, Hale L, Niu S, Yu G, Liu Y, et al. (2018) Soil organic matter availability and climate drive latitudinal patterns in bacterial diversity from tropical to cold temperate forests. Funct Ecol 32: 61–70.

[20] Wang J, Wang Y, He N, Ye Z, Chen C, Zang R, et al. (2020) Plant functional traits regulate soil bacterial diversity across temperate deserts. Sci Total Environ 715: 136976. doi: 10.1016/j.scitotenv.2020.136976 32023517

[21] Wang S, Zuo X, Zhao X, Awada T, Luo Y, Li Y, et al. (2018) Dominant plant species shape soil bacterial community in semiarid sandy land of northern China. Ecol Evol 8: 1693–1704 doi: 10.1002/ece3.3746 29435244 PMC5792618

[22] Harris JA (2003) Measurements of the soil microbial community for estimating the success of restoration. Eur J Soil Sci 54: 801–808.

[23] Philippot L, Raaijmakers JM, Lemanceau P, van der Putten WH (2013) Going back to the roots: The microbial ecology of the rhizosphere. Nat Rev Microbiol 11: 789–799. doi: 10.1038/nrmicro3109 24056930

[24] Ling L, Fu Y, Jeewani PH, Tang C, Pan S, Reid BJ, et al. (2021) Organic matter chemistry and bacterial community structure regulate decomposition processes in post-fire forest soils. Soil Biol Biochem 160: 108311.

[25] Delavaux CS, Weigelt P, Magnoli SM, Kreft H, Crowther TW, Bever JD (2022) Nitrogen-fixing symbiotic bacteria act as a global filter for plant establishment on islands. Commun Biol 5: 1209. doi: 10.1038/s42003-022-04133-x 36357567 PMC9649727

[26] Kiss E, Hegedüs B, Virágh M, Varga T, Merényi Z, Kószó T, et al. (2019) Comparative genomics reveals the origin of fungal hyphae and multicellularity. Nat Commun 10: 4080. doi: 10.1038/s41467-019-12085-w 31501435 PMC6733946

[27] De Beeck MO, Persson P, Tunlid A (2021) Fungal extracellular polymeric substance matrices–Highly specialized microenvironments that allow fungi to control soil organic matter decomposition reactions. Soil Biol Biochem 159: 108304.

[28] Liu L, Gunina A, Zhang F, Cui Z, Tian J (2023) Fungal necromass increases soil aggregation and organic matter chemical stability under improved cropland management and natural restoration. Sci Total Environ 858: 159953. doi: 10.1016/j.scitotenv.2022.159953 36368393

[29] Miller RM, Jastrow (2000) Mycorrhizal fungi influence soil structure. In: Kapulnik Y, Douds DD (eds) Arbuscular Mycorrhizas: Physiology and Function. Netherlands: Kluwer Academic Publishers. 3–18.

[30] Zan G, Wang C, Li F, Liu Z, Sun T (2023) Key data results and trend analysis of the sixth national survey on desertification and sandification. Forest resources management 1: 1–7.

[31] Li J, Han L, Liu Y, Zhang G, Wu (2018). Insights on historical expansions of desertification in the Hunlun Buir and Horqin Deserts of Northeast China. Ecol Indic 85: 944–950.

[32] Yan D (2010) Hulunbeier Sandy Land. Hohhot: Inner Mongolian Shantou University Press. 1–4.

[33] Gou F, Liang W, Sun S, Jin Z, Zhang W, Yan J (2021) Analysis of the desertification dynamics of sandy lands in northern China over the period 2000–2017. Geocarto Int 36: 1938–1959.

[34] Hijmans RJ, Cameron SE, Parra JL, Jones PG, Jarvis A (2005) Very high resolution interpolated climate surfaces for global land areas. Int J Climatol 25: 1965–1978.

[35] Lefroy RDB, Blair GJ, Strong WM (1993) Changes in soil organic-matter with cropping as measured by organic-carbon fractions and 13C natural isotope abundance. Plant Soil 155:399–402.

[36] Wei X, Hu Y, Peng P, Zhu Z, Atere GT, O’Donnell AG, et al. (2017) Effect of P stoichiometry on the abundance of nitrogen-cycle genes in phosphorus-limited paddy soil. Biol Fertil Soils 53:767–776.

[37] Lu R (1999) Methods of agrochemical soil analysis. Beijing: China Agricultural Science Press.

[38] Yu Y, Lee C, Kim J, Hwang S (2005) Group-specific primer and probe sets to detect methanogenic communities using quantitative real-time polymerase chain reaction. Biotechnol Bioeng 89: 670–679. doi: 10.1002/bit.20347 15696537

[39] White TJ, Bruns TD, Lee SB, Taylor JW, Innis MA, Gelfand DH, et al. (1990) Amplification and direct sequencing of fungal ribosomal RNA genes for phylogenetics. PCR Protocols 38: 315–322.

[40] W Zhang, Y Du (2018) Analysis of the succession of structure of the bacteria community in soil from long-term continuous cotton cropping in Xinjiang using high-throughput sequencing. Arch Microbiol 200: 653–662. doi: 10.1007/s00203-018-1476-4 29352369

[41] Martin M (2011) Cutadapt removes adapter sequences from high-throughput sequencing reads. EMBnet J 17: 10–12.

[42] Edgar RC, Haas BJ, Clemente JC, Quince C, Knight R (2011) UCHIME improves sensitivity and speed of chimera detection. Bioinformatics 15: 2194–2200. doi: 10.1093/bioinformatics/btr381 21700674 PMC3150044

[43] Pruesse E, Quast C, Knittel K, Fuchs BM, Ludwig W, Peplies J, et al. (2007) SILVA: a comprehensive online resource for quality checked and aligned ribosomal RNA sequence data compatible with ARB. Nucleic Acids Res 35:7188–7196. doi: 10.1093/nar/gkm864 17947321 PMC2175337

[44] Nilsson RH, Larsson KH, Taylor AFS, Bengtsson-Palme J, Jeppesen TS, Schigel D, et al. (2019) The UNITE database for molecular identification of fungi: handling dark taxa and parallel taxonomic classifications. Nucleic Acids Res 47: D259–d264. doi: 10.1093/nar/gky1022 30371820 PMC6324048

[45] Ursell LK, Robbins-Pianka A, Scott N, Gonzalez A, Knights D, Rideout JR, et al. (2015) Using QIIME to evaluate the microbial communities within hydrocarbon environments In: McGenity TJ, Timmis KN, Nogales Fernández B (eds) Hydrocarbon and Lipid Microbiology Protocols. Springer Protocols Handbooks. Springer, Berlin Heidelberg, Berlin, Heidelberg, 89–113.

[46] Clarke KR (1993) Non-parametric multivariate analyses of changes in community structure. Aust J Ecol 18: 117–143.

[47] Sickle JV (1997) Using mean similarity dendrograms to evaluate classifications. J Agr Biol Envir St 23: (1997) 70–88.

[48] Zapala MA, Schork NJ (2006) Multivariate regression analysis of distance matrices for testing associations between gene expression patterns and related variables. Proc Natl Acad Sci USA 103: 19430–19435. doi: 10.1073/pnas.0609333103 17146048 PMC1748243

[49] R Core Team, R: Alanguage and environment for statistical computing. RFoundation for Statistical Computing; Vienna, Austria (2017). http://www.R-project.org/

[50] ter Braak CJF, Smilauer P (2012) Canoco reference manual and user’s guide: software for ordination, version 5.0. Microcomputer Power, Ithaca.

[51] Gong X, Jarvie S, Zhang Q, Liu Q, Yan Y, Su N, et al. (2023) Community assembly of plant, soil bacteria, and fungi vary during the restoration of an ecosystem threatened by desertification. J Soil Sediment 23: 459–472.

[52] Lal R (2004) Soil carbon sequestration to mitigate climate change. Geoderma 123: 1–22.

[53] Su Y, Wang X, Yang R, Lee J (2010) Effects of sandy desertified land rehabilitation on soil carbon sequestration and aggregation in an arid region in China. J Environ Manage 91: 2109–2116. doi: 10.1016/j.jenvman.2009.12.014 20630649

[54] Yuan J, Ou Y, Zheng H, Xu W (2012) Effects of different grassland restoration approaches on soil properties in the southeastern Horqin sandy land, northern China. Appl Soil Ecol 61: 34–39.

[55] Li J, Awasthi MK, Zhu Q, Chen X, Wu F, Wu F, et al. (2021) Modified soil physicochemical properties promoted sequestration of organic and inorganic carbon synergistically during revegetation in desertified land. J Environ Chem Eng 9: 106331.

[56] Zhang Y, Chen L, Chen X, Tan M, Duan Z, Wu Z, et al. (2015) Response of soil enzyme activity to long-term restoration of desertified land. Catena 133: 64–70.

[57] Li Y, Chen Y, Wang X, Niu Y, Lian J (2017) Improvements in soil carbon and nitrogen capacities after shrub planting to stabilize sand dunes in China’s Horqin Sandy Land. Sustainability 9: 662.

[58] Xiao J, Xiong K (2022) A review of agroforestry ecosystem services and its enlightenment on the ecosystem improvement of rocky desertification control. Sci Total Environ 852: 158538. doi: 10.1016/j.scitotenv.2022.158538 36067859

[59] Qi Y, Yang F, Shukla MK, Pu J, Chang Q (2015) Desert soil properties after thirty years of vegetation restoration in Northern Shaanxi Province of China. Arid Land Res Manag 29: 454–472.

[60] Lei X, Shen Y, Zhao J, Huang J, Wang H, Yu Y, et al. (2023) Root exudates mediate the processes of soil organic carbon input and efflux. Plants 12: 630. doi: 10.3390/plants12030630 36771714 PMC9919716

[61] Angelova VR, Akova VI, Artinova NS (2013) The effect of organic amendments on soil chemical characteristics. Bulg J Agric Sci 19: 958–971.

[62] Sun S, Li S, Avera BN, Strahm BD, Badgley BD (2017) Soil bacterial and fungal communities show distinct recovery patterns during forest ecosystem restoration. Appl Environ Microb 83: e00966–17. doi: 10.1128/AEM.00966-17 28476769 PMC5494632

[63] Čaušević S, Dubey M, Morales M, Salazar G (2024) Niche availability and competitive loss by facilitation control proliferation of bacterial strains intended for soil microbiome interventions. Nat Commun 15: 2557. doi: 10.1038/s41467-024-46933-1 38519488 PMC10959995

[64] Fernandez AL, Sheaffer CC, Wyse DL, Staley C, Gould TJ, Sadowsky MJ (2016) Associations between soil bacterial community structure and nutrient cycling functions in long-term organic farm soils following cover crop and organic fertilizer amendment. Sci Total Environ 566–567: 949–959. doi: 10.1016/j.scitotenv.2016.05.073 27288977

[65] Meena M, Swapnil P, Zehra A, Aamir M (2017) Beneficial microbes for disease suppression and plant growth promotion. In Plant-Microbe Interactions in Agro-Ecological Perspectives; Springer: Berlin/Heidelberg, Germany, 395–432.

[66] Adesemoye AO, Kloepper JW (2009) Plant–microbes interactions in enhanced fertilizer-use efficiency. Appl Microbial Biot 85: 1–12. doi: 10.1007/s00253-009-2196-0 19707753

[67] Mitra D, Mondal R, Khoshru B, Senapati A, Radha TK, Mahskur B, et al. (2022) Actinobacteria enhanced plant growth, nutrient acquisition, and crop protection: Advances in soil, plant, and microbial multifactorial interactions. Pedosphere 32: 149–170.

[68] García-Fraile P, Benada O, Cajthaml T, Baldrian P, Lladó S (2016) Terracidiphilus gabretensis gen. nov., sp. nov., an abundant and active forest soil acidobacterium important in organic matter transformation. Appl Environ Microb 82: 3353–15. doi: 10.1128/AEM.03353-15 26546425 PMC4711116

[69] Dai H, Zang H, Zhao, Qian X Y, Liu K (2019) Linking bacterial community to aggregate fractions with organic amendments in a sandy soil. Land Degrad Dev 30: 1828–1839.

[70] Yang L, Barnard R, Kuzyakov Y, Tian J (2021) Bacterial communities drive the resistance of soil multifunctionality to land-use change in karst soils. Eur J Soil Biol 104: 103313.

[71] Filippidou S, Wunderlin T, Junier T (2016) A combination of extreme environmental conditions favor the prevalence of endospore-forming firmicutes. Front Microbiol 7: 1707. doi: 10.3389/fmicb.2016.01707 27857706 PMC5094177

[72] Wang S, Zuo X, Zhao X, Li Y, Zhou X, Lv P (2016) Responses of soil fungal community to the sandy grassland restoration in Horqin Sandy Land, northern China. Environ Monit Assess 188: 21. doi: 10.1007/s10661-015-5031-3 26661957

[73] Brussaard L (1997) Biodiversity and ecosystem functioning in soil. Ambio 26: 563–570. 10.1007/s13280-021-01507-z PMC8116420 33713290

[74] Read DJ, Perez-Moreno J (2003) Mycorrhizas and nutrient cycling in ecosystems–a journey towards relevance? New phytol 157: 475–492. doi: 10.1046/j.1469-8137.2003.00704.x 33873410

[75] Liu Y, Havrilla CA, Jia C, Liu X, Wu G (2021) Litter crusts enhance soil nutrients through bacteria rather than fungi in sandy ecosystems. Catena 204: 105413.

[76] Berrios L, Bogar GD, Bogar LM, Venturini AM (2024) Ectomycorrhizal fungi alter soil food webs and the functional potential of bacterial communities. Msystems 9: e00369–24. doi: 10.1128/msystems.00369-24 38717159 PMC11237468

[77] Andreote FD, P e Silva MC (2017) Microbial communities associated with plants: learning from nature to apply it in agriculture. Curr Opin Microbiol 37: 29–34. doi: 10.1016/j.mib.2017.03.011 28437663

[78] Broughton LC, Gross KL (2000) Patterns of diversity in plant and soil microbial communities along a productivity gradient in a Michigan old-field. Oecologia 125: 420–427. doi: 10.1007/s004420000456 28547337

[79] Shanmugam SG, Kingery WL (2018) Changes in soil microbial community structure in relation to plant succession and soil properties during 4000 years of pedogenesis. Eur J Soil Biol 88: 80–88.

[80] Chen Y, Xu T, Veresoglou S, Hu H, Hao Z, Hu Y, et al. (2017) Plant diversity represents the prevalent determinant of soil fungal community structure across temperate grasslands in northern China. Soil Biol Biochem 110: 12–21.

[81] Shi S, Richardson AE, O’Callaghan M, Firestone M, Condron L (2013) Challenges in assessing links between root exudates and the structure and function of soil microbial communities. Mol Microb Ecol Rhizosphere 2: 125–135.

[82] Seaton FM, George PBL, Lebron I, Jones DL (2020) Soil textural heterogeneity impacts bacterial but not fungal diversity. Soil Biol Biochem 144: 107766.

[83] Qi D, Wieneke X, Tao J, Zhou X, Desilva U (2018) Soil pH is the primary factor correlating with soil microbiome in karst rocky desertification regions in the Wushan County, Chongqing, China. Front Microbiol 9: 1027. 29896164 10.3389/fmicb.2018.01027 PMC5987757

[84] Zhalnina K, Dias R, de Quadros PD (2015) Soil pH determines microbial diversity and composition in the park grass experiment. Microb Ecol 69: 395–406. doi: 10.1007/s00248-014-0530-2 25395291

[85] Rengel Z, Marschner P (2005) Nutrient availability and management in the rhizosphere: exploiting genotypic differences. New Phytol 168: 305–312. doi: 10.1111/j.1469-8137.2005.01558.x 16219070

[86] Shaheen SM, Tsadilas CD, Rinklebe J (2013) A review of the distribution coefficients of trace elements in soils: Influence of sorption system, element characteristics, and soil colloidal properties. Adv Colloid Interfac 201–202: 43–56. doi: 10.1016/j.cis.2013.10.005 24168932

[87] Rousk J, Bååth E, Brookes PC, Lauber CL, Lozupone C, Caporaso JG, et al. (2010) Soil bacterial and fungal communities across a pH gradient in an arable soil. ISME J 4: 1340–1351. doi: 10.1038/ismej.2010.58 20445636

[88] Philippot L, Griffiths BS, Langenheder S (2021) Microbial community resilience across ecosystems and multiple disturbances. Microbiol Mol Biol Rev 85: e00026–20. doi: 10.1128/MMBR.00026-20 33789927 PMC8139522

