# Supplementary material for: Soil bacterial and fungal diversity and composition respond differently to desertified system restoration
Source: PLoS One. 2025 Jan 6;20(1):e0309188. doi: 10.1371/journal.pone.0309188 (PMC11703004; doi:10.1371/journal.pone.0309188)
Supplement: S1 Table — (DOCX) [file pone.0309188.s001.docx]

| Sample | pH | NO_3_^—^N  (mg kg^-1^) | NH_4_^+^-N  (mg kg^-1^) | Clay  (%) | SOC  (g kg^-1^) | TN  (g kg^-1^) | PR  (species/m^2^) | PB  (g/m^2^) |
| --- | --- | --- | --- | --- | --- | --- | --- | --- |
| MD1 | 7.19 | 2.97 | 4.35 | 2.10 | 0.52 | 0.12 | 2.00 | 3.09 |
| MD2 | 7.24 | 2.25 | 4.40 | 6.50 | 0.68 | 0.18 | 2.00 | 7.89 |
| MD3 | 7.32 | 2.65 | 4.75 | 4.79 | 0.66 | 0.09 | 2.00 | 22.75 |
| MD4 | 8.11 | 2.4 | 5.05 | 3.50 | 0.54 | 0.16 | 1.50 | 4.46 |
| MD5 | 8.44 | 2.71 | 5.15 | 8.58 | 0.55 | 0.09 | 2.00 | 16.95 |
| MD6 | 7.85 | 2.3 | 4.85 | 2.50 | 0.50 | 0.16 | 2.50 | 8.20 |
| MD7 | 8.15 | 2.55 | 4.55 | 6.29 | 0.63 | 0.14 | 1.50 | 6.32 |
| MD8 | 7.52 | 2.44 | 4.65 | 3.59 | 0.65 | 0.07 | 0.00 | 0.00 |
| MD9 | 7.69 | 2.36 | 4.45 | 9.48 | 0.63 | 0.13 | 0.00 | 0.00 |
| MD10 | 7.56 | 2.48 | 4.75 | 5.30 | 0.75 | 0.20 | 0.00 | 0.00 |
| MD11 | 7.53 | 2.12 | 4.50 | 4.50 | 0.61 | 0.19 | 0.00 | 0.00 |
| MD12 | 7.54 | 2.47 | 4.25 | 4.29 | 0.87 | 0.13 | 0.50 | 1.04 |
| MD13 | 7.31 | 2.48 | 5.10 | 3.60 | 0.63 | 0.11 | 0.00 | 0.00 |
| MD14 | 8.44 | 2.62 | 6.28 | 8.80 | 1.25 | 0.21 | 0.00 | 0.00 |
| MD15 | 8.41 | 1.82 | 4.75 | 6.09 | 0.72 | 0.14 | 1.50 | 3.38 |
| FD1 | 7.11 | 2.45 | 5.85 | 3.49 | 1.33 | 0.27 | 5.00 | 21.81 |
| FD2 | 7.03 | 2.94 | 5.85 | 10.99 | 1.11 | 0.26 | 6.00 | 23.70 |
| FD3 | 7.38 | 2.81 | 6.35 | 3.20 | 1.78 | 0.23 | 7.50 | 32.52 |
| FD4 | 6.78 | 3.09 | 5.60 | 17.17 | 2.15 | 0.21 | 6.50 | 22.95 |
| FD5 | 7.53 | 7.06 | 6.75 | 7.10 | 2.28 | 0.22 | 5.00 | 20.00 |
| FD6 | 7.56 | 5.02 | 6.1 | 14.27 | 2.09 | 0.39 | 5.50 | 16.34 |
| FD7 | 7.47 | 6.26 | 6.8 | 9.30 | 4.10 | 0.40 | 6.00 | 41.79 |
| FD8 | 7.44 | 6.63 | 7.20 | 11.19 | 4.06 | 0.42 | 6.00 | 25.88 |
| FD9 | 7.22 | 7.39 | 8.03 | 18.86 | 4.39 | 0.47 | 7.00 | 20.10 |
| FD10 | 6.66 | 6.39 | 7.50 | 23.48 | 8.30 | 0.94 | 6.50 | 34.54 |
| FD11 | 6.52 | 8.05 | 9.05 | 11.90 | 7.30 | 0.82 | 10.00 | 36.52 |
| FD12 | 7.03 | 4.99 | 8.45 | 25.05 | 8.55 | 1.00 | 9.00 | 29.18 |
| FD13 | 7.24 | 7.45 | 4.94 | 12.10 | 4.13 | 2.13 | 7.5 | 30.90 |
| FD14 | 6.99 | 4.85 | 6.70 | 16.37 | 4.50 | 0.48 | 9.0 | 28.05 |
| FD15 | 7.6 | 9.05 | 6.45 | 6.39 | 4.06 | 0.46 | 6.0 | 29.74 |

**S1 Table: Detailed vegetation and soil properties of** **mobile and fixed dunes**

Note: MB: Moblie dunes; FD: Fixed dunes. NH_4_^+^-N, NO_3_*^˗^*-N, TC, TN represents the abbreviations of soil ammonium nitrogen, soil nitrate nitrogen, soil total carbon, soil total nitrogen, while PB and PR represents plant aboveground biomass and plant species richness, respectively.
