# Supplementary material for: Soil bacterial and fungal diversity and composition respond differently to desertified system restoration
Source: PLoS One. 2025 Jan 6;20(1):e0309188. doi: 10.1371/journal.pone.0309188 (PMC11703004; doi:10.1371/journal.pone.0309188)
Supplement: S2 Table — The alpha diversity indices were based on the OTU richness (Chao 1 index) and diversity (Shannon diversity) indices. (DOCX) [file pone.0309188.s002.docx]

**S2 Table: Soil bacterial and fungal alpha-diversity indices in mobile and fixed dunes. The alpha diversity indices were based on the OTU richness (Chao 1 index) and diversity (Shannon diversity) indices.**

|  | Bacteria | |  | Fungi | |
| --- | --- | --- | --- | --- | --- |
| Sample | Chao1 | Shannon |  | Chao1 | Shannon |
| MD1 | 2546.723 | 9.173 |  | 245.267 | 1.505 |
| MD2 | 3224.133 | 7.037 |  | 180.375 | 2.747 |
| MD3 | 3185.196 | 8.736 |  | 202.222 | 2.510 |
| MD4 | 2308.024 | 7.470 |  | 202.071 | 4.272 |
| MD5 | 2375.091 | 7.192 |  | 311.389 | 3.074 |
| MD6 | 2330.171 | 7.653 |  | 284.857 | 2.843 |
| MD7 | 1998.605 | 7.406 |  | 373.950 | 3.353 |
| MD8 | 2881.017 | 8.101 |  | 509.514 | 3.520 |
| MD9 | 1222.059 | 7.107 |  | 202.977 | 2.900 |
| MD10 | 3247.158 | 8.160 |  |  |  |
| MD11 | 2225.280 | 8.059 |  |  |  |
| MD12 | 2022.251 | 5.915 |  |  |  |
| MD13 | 2277.335 | 8.659 |  |  |  |
| MD14 | 814.551 | 3.878 |  |  |  |
| MD15 | 1758.692 | 6.767 |  |  |  |
| FD1 | 4038.910 | 9.681 |  | 156.652 | 3.162 |
| FD2 | 3180.845 | 9.236 |  | 162.875 | 3.010 |
| FD3 | 3657.009 | 9.593 |  | 155.235 | 2.472 |
| FD4 | 3076.114 | 9.198 |  | 179.529 | 2.923 |
| FD5 | 2831.587 | 9.005 |  | 182.610 | 3.687 |
| FD6 | 2826.057 | 8.775 |  | 312.776 | 3.794 |
| FD7 | 3017.290 | 9.203 |  | 158.724 | 2.742 |
| FD8 | 1060.483 | 5.402 |  | 176.625 | 2.386 |
| FD9 | 3310.041 | 9.266 |  | 186.477 | 2.513 |
| FD10 | 3335.638 | 9.308 |  | 141.391 | 2.829 |
| FD11 | 3796.026 | 9.349 |  | 164.800 | 2.356 |
| FD12 | 3743.093 | 9.617 |  | 188.865 | 2.683 |
| FD13 | 2978.366 | 9.023 |  |  |  |
| FD14 | 3635.005 | 9.664 |  |  |  |
| FD15 | 3395.771 | 9.115 |  |  |  |

Note: MB: Moblie dunes; FD: Fixed dunes.
