# Supplementary material for: Soil bacterial and fungal diversity and composition respond differently to desertified system restoration
Source: PLoS One. 2025 Jan 6;20(1):e0309188. doi: 10.1371/journal.pone.0309188 (PMC11703004; doi:10.1371/journal.pone.0309188)
Supplement: S3 Table — (DOCX) [file pone.0309188.s003.docx]

**S3 Table: Relative abundance greater than 1% of soil bacterial phyla in mobile and fixed dunes.**

| phylum | Proteobacteria | Firmicutes | Actinobacteria | Bacteroidetes | Acidobacteria | Gemmatimonadetes | Chloroflexi | Cyanobacteria | Others |
| --- | --- | --- | --- | --- | --- | --- | --- | --- | --- |
| MD1 | 0.303971 | 0.14479 | 0.15084 | 0.187375 | 0.03865 | 0.013588 | 0.065513 | 0.003583 | 0.09169 |
| MD2 | 0.454869 | 0.340545 | 0.076458 | 0.035948 | 0.026139 | 0.015213 | 0.01911 | 0.001938 | 0.02978 |
| MD3 | 0.373008 | 0.187199 | 0.116596 | 0.123507 | 0.033951 | 0.01212 | 0.034616 | 0.054548 | 0.064455 |
| MD4 | 0.508674 | 0.23139 | 0.11221 | 0.041234 | 0.030936 | 0.022418 | 0.019756 | 0.001488 | 0.031895 |
| MD5 | 0.465971 | 0.328934 | 0.078259 | 0.048714 | 0.023241 | 0.013627 | 0.015526 | 0.001038 | 0.02469 |
| MD6 | 0.480988 | 0.199279 | 0.177546 | 0.02561 | 0.035713 | 0.03072 | 0.020715 | 0.001077 | 0.028351 |
| MD7 | 0.54654 | 0.262776 | 0.051239 | 0.057466 | 0.016799 | 0.009026 | 0.007146 | 0.019677 | 0.02933 |
| MD8 | 0.466911 | 0.26628 | 0.12942 | 0.045561 | 0.030152 | 0.013314 | 0.01631 | 0.00464 | 0.027411 |
| MD9 | 0.29048 | 0.458276 | 0.016897 | 0.173239 | 0.003779 | 0.001468 | 0.001782 | 0.036574 | 0.017504 |
| MD10 | 0.448702 | 0.19225 | 0.207973 | 0.03119 | 0.041998 | 0.035987 | 0.017798 | 0.002056 | 0.022046 |
| MD11 | 0.514508 | 0.208678 | 0.087598 | 0.040451 | 0.043525 | 0.028273 | 0.014606 | 0.001527 | 0.060833 |
| MD12 | 0.507616 | 0.343404 | 0.077907 | 0.022693 | 0.015429 | 0.014685 | 0.006305 | 0.000666 | 0.011297 |
| MD13 | 0.391804 | 0.294122 | 0.083643 | 0.134785 | 0.017563 | 0.006226 | 0.006833 | 0.01674 | 0.048283 |
| MD14 | 0.778439 | 0.010984 | 0.190116 | 0.004836 | 0.002369 | 0.008145 | 0.003015 | 0.000215 | 0.00188 |
| MD15 | 0.680405 | 0.088401 | 0.090477 | 0.065102 | 0.016721 | 0.018855 | 0.010964 | 0.001155 | 0.02792 |
| FD1 | 0.326761 | 0.075694 | 0.334926 | 0.027705 | 0.07311 | 0.070858 | 0.047226 | 0.012942 | 0.030779 |
| FD2 | 0.370306 | 0.115088 | 0.281866 | 0.035752 | 0.062928 | 0.044876 | 0.032482 | 0.015781 | 0.040921 |
| FD3 | 0.346027 | 0.086541 | 0.320848 | 0.029859 | 0.066942 | 0.063633 | 0.039139 | 0.013157 | 0.033853 |
| FD4 | 0.465619 | 0.069135 | 0.222462 | 0.04237 | 0.077163 | 0.03585 | 0.040392 | 0.003896 | 0.043114 |
| FD5 | 0.447742 | 0.046501 | 0.262423 | 0.044465 | 0.068959 | 0.038454 | 0.044582 | 0.001625 | 0.045248 |
| FD6 | 0.464561 | 0.134472 | 0.201981 | 0.053628 | 0.058405 | 0.021439 | 0.030759 | 0.001625 | 0.033128 |
| FD7 | 0.478169 | 0.099287 | 0.200474 | 0.034421 | 0.060207 | 0.034186 | 0.057583 | 0.001488 | 0.034186 |
| FD8 | 0.032228 | 0.752124 | 0.012374 | 0.182108 | 0.002467 | 0.000881 | 0.001371 | 0.000117 | 0.016329 |
| FD9 | 0.46973 | 0.086228 | 0.241199 | 0.030975 | 0.0605 | 0.040941 | 0.041763 | 0.000979 | 0.027685 |
| FD10 | 0.446235 | 0.021459 | 0.251674 | 0.089576 | 0.081196 | 0.036457 | 0.02978 | 0.0028 | 0.040823 |
| FD11 | 0.461311 | 0.045601 | 0.262247 | 0.046364 | 0.07869 | 0.037514 | 0.026706 | 0.001468 | 0.040099 |
| FD12 | 0.405686 | 0.02888 | 0.252575 | 0.047637 | 0.118416 | 0.034362 | 0.044367 | 0.023476 | 0.044602 |
| FD13 | 0.466597 | 0.10892 | 0.206504 | 0.028155 | 0.063809 | 0.041411 | 0.039022 | 0.00141 | 0.044171 |
| FD14 | 0.415573 | 0.060951 | 0.279027 | 0.035615 | 0.095802 | 0.030407 | 0.040451 | 0.001782 | 0.040392 |
| FD15 | 0.500529 | 0.034225 | 0.218604 | 0.040627 | 0.076888 | 0.052688 | 0.028429 | 0.003015 | 0.044994 |

Note: MB: Moblie dunes; FD: Fixed dunes.
