# Supplementary material for: Soil bacterial and fungal diversity and composition respond differently to desertified system restoration
Source: PLoS One. 2025 Jan 6;20(1):e0309188. doi: 10.1371/journal.pone.0309188 (PMC11703004; doi:10.1371/journal.pone.0309188)
Supplement: S4 Table — (DOCX) [file pone.0309188.s004.docx]

**S4 Table: Relative abundance greater than 1% of soil fungal phyla in mobile and fixed dunes.**

| Taxonomy | Ascomycota | Basidiomycota | Rozellomycota | Others |
| --- | --- | --- | --- | --- |
| MD1 | 0.991765 | 0.002347 | 0.000000 | 0.005888 |
| MD2 | 0.994688 | 0.000225 | 0.000000 | 0.005087 |
| MD3 | 0.970628 | 0.000337 | 0.000000 | 0.029035 |
| MD4 | 0.905981 | 0.000646 | 0.000000 | 0.093372 |
| MD5 | 0.373489 | 0.000675 | 0.000000 | 0.625836 |
| MD6 | 0.349275 | 0.000675 | 0.000000 | 0.650051 |
| MD7 | 0.893431 | 0.073767 | 0.004863 | 0.027939 |
| MD8 | 0.945612 | 0.034937 | 0.002066 | 0.017384 |
| MD9 | 0.992355 | 0.004849 | 0.000000 | 0.002797 |
| FD1 | 0.976643 | 0.000731 | 0.000000 | 0.022626 |
| FD2 | 0.977908 | 0.000632 | 0.000000 | 0.021460 |
| FD3 | 0.975490 | 0.000267 | 0.000000 | 0.024243 |
| FD4 | 0.993718 | 0.001181 | 0.000141 | 0.004961 |
| FD5 | 0.990472 | 0.000267 | 0.000028 | 0.009233 |
| FD6 | 0.984485 | 0.004610 | 0.000014 | 0.010892 |
| FD7 | 0.993001 | 0.000323 | 0.000000 | 0.006675 |
| FD8 | 0.901540 | 0.000436 | 0.000000 | 0.098024 |
| FD9 | 0.929507 | 0.000436 | 0.000000 | 0.070057 |
| FD10 | 0.993802 | 0.000267 | 0.000000 | 0.005931 |
| FD11 | 0.995615 | 0.000394 | 0.000000 | 0.003991 |
| FD12 | 0.995180 | 0.000225 | 0.000000 | 0.004596 |

Note: MB: Moblie dunes; FD: Fixed dunes.
